# Chickpea Aquafaba-Based Emulsions as a Fat Replacer in Pound Cake: Impact on Cake Properties and Sensory Analysis

**DOI:** 10.3390/foods11162484

**Published:** 2022-08-17

**Authors:** Graziele Grossi Bovi Karatay, Ana Paula Rebellato, Caroline Joy Steel, Miriam Dupas Hubinger

**Affiliations:** Department of Food Engineering and Technology, School of Food Engineering, University of Campinas (UNICAMP), Monteiro Lobato Street, 80, Campinas 13083-862, Brazil

**Keywords:** pulses, saturated fat alternatives, bakery products, reformulation

## Abstract

This study evaluates the use of chickpea aquafaba (CA)-based emulsions as a potential substitute for palm oil (PO), using pound cake as a case study. The CA was characterized in terms of pH (6.38 ± 0.01), density (1.02 g mL^−1^ ± 0.01), color, total soluble solids (6.3 ± 0.2 °Bx), total solids (5.7 ± 0.2%), thermal properties through DSC, and apparent viscosity (2.5 cPa·s^−1^ ± 0.02 at 300 s^−1^). Emulsions containing 35, 30, and 25% of CA were produced and applied to cake formulation C1, C2, and C3, respectively. The cake batter was evaluated in terms of apparent density (0.87–1.04 g^1^ cm^−3^), rheology, and pH (6.6–6.8). The cakes were evaluated for specific volume, baking loss (8.9–9.5%), color, and symmetry index on day 1, and firmness, water activity (aw), and moisture content (%), after 14 days of storage. The cakes produced with the emulsions were found to have slightly higher specific volume (2.3 cm^3^ g^−1^) when compared to the control (C4) produced with PO (2.2 cm^3^ g^−1^). The moisture and aw decreased and firmness increased during storage. In terms of formulation (i.e., day 1 for C1, C2, C3, and C4), there was no significant difference for moisture. As for aw, the C4 (0.90) was significantly different from the cakes produced with emulsions (0.91–0.92). The results from the sensory evaluation, carried out with 120 panelists, showed no statistically significant difference between C3 and C4 for the attributes of aroma, color, texture, flavor, and overall impression. Based on our results, it appears that the CA-based emulsions have the potential to replace PO in pound-cake recipes, reducing total and saturated fat.

## 1. Introduction

The rise in cardiovascular diseases over the last few decades has been linked to the increased intake of saturated fatty acids (SFAs) and *trans* fatty acids (TFAs) in the human diet [1,2]. This has led to efforts, both from the scientific and industrial communities, to find alternatives to decrease the use of SFAs and TFAs in food products. Many countries have already called for the total removal of TFAs, as well as for the reduction in SFAs from processed foods [3,4,5]. Such a removal/reduction is challenging, since the SFAs and TFAs are responsible for providing taste, shelf-life, texture, and structure to a wide variety of processed food products [6,7]. The oil structuring approaches are a highly relevant area of research to address these challenges, as they enable the replacement of SFAs and TFAs with polyunsaturated fatty acids (PUFAs).

Oil structuring, also known as oleogelation, is the process of transforming liquid oil into structured systems through different production methods, with direct and indirect approaches [7,8]. In recent years, oil structuring has gained a lot of attention due to the possibility of forming different lipid-based structures with desired rheological properties. For instance, different types of structured oils, such as oleogels, bigels, and advanced emulsions (i.e., high internal phase emulsions (HIPEs) and Pickering emulsions (HIPPEs), emulgels, microgels, among others) can be produced by using a wide variety of structuring agents (e.g., waxes, ethyl cellulose, shellac, sterols, monoacylglycerols, polysaccharides, and proteins). In this context, the ingredient engineering can contribute to the production of diverse lipid-based systems with desired technological functions for specific food matrixes, aiming at the replacement of TFAs and SFAs by healthier fat types and, consequently, aggregating benefits to consumers’ health.

Pulses, such as lentils, beans, chickpeas, and peas, are suitable candidates to be used as structuring agents for vegetable liquid oils (rich in PUFAs), due to their functional properties. In addition, their use is in line with the trend driven by consumer concerns towards decreasing synthetic ingredients in food products [9]. Moreover, pulses have been recognized as a prospective economically and environmentally sustainable substitute for animal proteins due to their high protein content, within the range of 17–30% [10]. As compared to other plant-based sources, such as soybeans, pulse proteins are an appealing substitute as they are usually not genetically modified and have not been identified as a potential allergen [11].

The heat processing of pulse seeds in water, by canning or boiling, yields a gelatinous liquid by-product called aquafaba. Aquafaba is a nutrient-rich ingredient with great potential for the replacement of animal-based proteins (e.g., eggs), due to its emulsifying and foaming properties [12]. Chickpea aquafaba (CA) has gained considerable attention in recent years and has mainly been tested for its foaming and emulsifying properties [13,14,15]. In fact, CA has been tested as an egg replacer in distinct foodstuffs, such as cakes [16,17], mayonnaise [18,19], and meringues [20,21]. However, CA has not yet been proposed and applied as a potential replacer for the SFAs and TFAs in cakes.

Cakes are a worldwide known product with great acceptance among customers all over the world [22]. Based on the preparation method, there are two basic types of cakes: (i) foamed (e.g., sponge, and chiffon), which rely on foam development and stability for their structure; and (ii) shortened (e.g., pound cake), which rely on the aeration of fat during batter processing [23]. The shortened-style cakes, also known as batter cakes, can be prepared by a multi-stage or single mixing approach [24]. In the multi-stage mixing process using the creaming method, sugar and fat are initially beaten together yielding a cream in which air is incorporated into the fat [25]. After the creaming stage, the eggs are included, followed by the alternate incorporation of milk and flour [23]. The final cake batter is composed of a multi-phase assembly with static gas cells trapped in the fat phase and all the other ingredients are dispersed or dissolved in the liquid phase [25].

The World Health Organization (WHO) warns about the need to reduce the consumption of SFAs and recommends eliminating the use of TFAs by the food industry. In addition, the consumer demands for healthier foods are constantly increasing and this pushes the market in the direction of the expansion of healthier (e.g., low-fat) food products. Following this tendency, many of the food product formulations need to be adapted, aiming at the use of lower and/or healthier fats. The replacement of the fat should at least preserve, or ideally even improve, the sensory attributes of the foodstuff. Therefore, it is necessary to apply potential SAFA and TFA substitutes in a food matrix and evaluate their feasibility in terms of the impact on product quality properties and sensory evaluation and acceptance to effectively assess their ability to act as a fat replacer. In this context, this study evaluates the potential of using CA-based conventional emulsions and HIPEs as a substitute for palm oil in a pound cake formulation, with respect to their effects on the technological and sensory characteristics. In addition, this study characterized CA from a Brazilian cultivar (BRS Aleppo) in terms of pH, density, color, total soluble solids (TSS), total solids (%), thermal properties through differential scanning calorimetry (DSC), and apparent viscosity.

## 2. Material and Methods

### 2.1. Material

The chickpeas (i.e., Kabuli type and BRS Aleppo cultivar), were kindly donated by Embrapa Vegetables (Brasília, DF, Brazil) and frozen until analysis. The canola oil (purity 100%; Seara Alimentos S.A., Gaspar, SC, Brazil), was acquired from a regional supermarket (Dalben, Campinas, SP, Brazil). The wheat flour type 1 (76% carbohydrate (m/m), 10% protein (m/m), 1.4% total fat (m/m), and 2.8% fiber (m/m) (Farinha Dona Benta; J. Macêdo S.A., Fortaleza, CE, Brazil)); the corn starch (85% carbohydrate (m/m), 0% protein (m/m), 0% total fat (m/m), and 0% fiber (m/m) (Maizena^®^; Unilever Brazil, Garanhuns, PE, Brazil)); the refined sugar (100% carbohydrate (m/m) (Colombo Agroindústria S/A, Ariranha, SP, Brazil)), the baking powder (Dr. Oetker Brazil, São Paulo, SP, Brazil), and the whole milk, 3% fat (Italac, Corumbaíba, GO, Brazil) were all obtained from a local supermarket (Carrefour, Campinas, SP, Brazil). The refined palm oil (Al Lette P39 LT, total fat 100%, of which 51% is saturated fat, 0.5% is *trans*-fat, 39% is monounsaturated fat and 39% is poli-unsaturated fat) was donated by Cargill Brazil (Mairinque, SP, Brazil). The whole, liquid pasteurized eggs were bought from Maxxi Ovos (Shinoda Alimentos LTDA., Indaiatuba, SP, Brazil). The calcium propionate (Alamar Tecno-Cientifica Ltd., Diadema, SP, Brazil), sodium chloride, citric acid, extra hydrated neutral alcohol, sorbic acid, propylene glycol (Dinâmica Química Contemporânea, Indaiatuba, SP, Brazil) were purchased from Sinergia Científica (Campinas, SP, Brazil).

### 2.2. Chickpea Aquafaba (CA) Production

The chickpea seeds were prepared as in [14]. In short, the chickpea seeds were pre-soaked (16 h at 4 °C) at a ratio of chickpea (C): distilled water (DW) of 1:3 (*w*/*v*). The pre-soaked seeds were then cooked in a pressure cooker (Instant Pot^®^ 7 in 1 multi-use programmable pressure cooker, IPDUO60 V2, 6 quart/liters) at 115–118 °C for 30 min at a ratio C:DW of 1:2. The cooked seeds were then left inside the pressure cooker for 6 h to cool down, followed by the aquafaba separation from the cooked chickpeas using a strainer. The aquafaba was then frozen until use. The proximate composition of CA consisted of 94.4% (moisture), 3.4% (carbohydrate), 1.2% (protein), 0.5% (ash), 0.5% (fiber), and 0.1% (fat), as determined in a previous study from our research group [14].

### 2.3. CA Characterization 

#### 2.3.1. Physicochemical Characteristics

The CA pH was determined in a pH Lab meter (model 827; Metrohm, Herisau, Switzerland) and the density was measured with a manual pycnometer. The density and pH measurements were performed in three replicates. The CA color was assessed in terms of L*, a*, and b* in sextuplicate by instrumental colorimetry (UltraScan VIS, Hunterlab, Reston, VA, USA). The total soluble solids (TSS) were quantified using a digital refractometer (DR-A1, Atago, Ribeirão Preto, SP, Brazil) and expressed as °Bx. The CA total solids’ content (%) was evaluated until a constant weight was reached in an infrared moisture analyzer (model MOC63u; Shimadzu, Japan) operating at 105 °C.

#### 2.3.2. Rheological Measurements

The CA rheological measurements were performed on an AR 1500 ex (TA Instruments, New Castle, PA, USA), using a 2° stainless-steel cone and plate (40 mm diameter and 47 µm gap).

The flow curves, in terms of shear rate versus shear stress, were performed with shear rate values ranging from 0 to 300 s^−1^, with three consecutive ramps. The records from the third flow curve were used to fit the power law model, Equation (1):

(1) $$\sigma =k\;\times \;\gamma^{n}$$

where σ is the shear stress (Pa); k is the flow consistency index (Pa·s^n^); γ is the shear rate (s^−1^); and n is the flow behavior index (dimensionless).

#### 2.3.3. Thermal Properties

The thermal properties through differential scanning calorimeter (DSC) (DSC1, Mettler Toledo, Schwerzenbach, Switzerland) were used to carry out the thermal analysis of the CA. A small portion of the freeze-dried CA (9.5520 mg) was collected and weighed in an aluminum pan (40 µL), using a microanalytical balance (XPR2; Mettler Toledo, Schwerzenbach, Switzerland). The pan was then sealed with a lid, perforated, and taken to the equipment for analysis. An empty pan was used as a reference and the analysis was performed in a heating ramp from 20 to 200 °C at 10 °C min^−1^, with a continuous purge of dry nitrogen gas of 50 mL min^−1^, according to the procedure described by [26].

### 2.4. Chickpea Aquafaba Based Emulsions Preparation

The canola oil (CO) was used as the oil phase and the CA as the aqueous phase. The conventional type emulsions, E1 (35% CA and 65% CO) and E2 (30% CA and 70% CO), and a high internal phase emulsion (HIPE), E3 (25% CA and 75% CO) were prepared using a rotor stator device (Ultraturrax^®^ T18 basic; IKA^®^-Werke GmbH & Co., KG, Staufen, Germany) operating at 15,500 rpm for 1 min, as prepared in [14].

### 2.5. Cake Production

**Figure 1:**
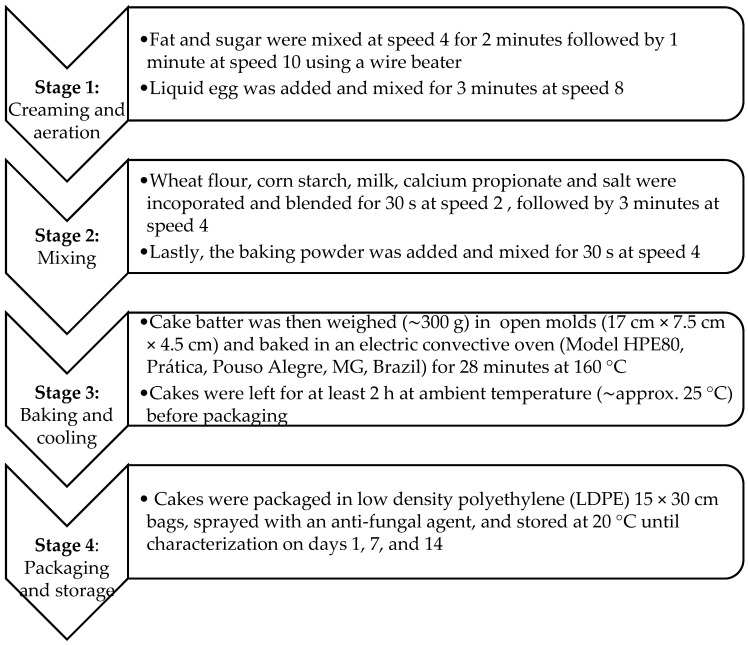
Flow diagram of the cake preparation multi-stage process. The anti-fungal agent consisted of 400 mL extra hydrated neutral alcohol, 100 mL deionized water, 1.35 g citric acid, 4.25 g sorbic acid, and 3.75 g propylene glycol.

The reference batter recipe was a pound cake recipe adapted from (Table 1) [27]. The four cake formulations containing the different types of fat, namely E1, E2, E3, and the palm oil were prepared using a Kitchen Aid mixer (Professional 600 6 QT; St. Joseph, MI, USA). The cake nomenclature was based on the type of fat added to each formulation and was as follows: C1–cake containing fat E1; C2–cake containing fat E2; C3–cake containing fat E3; and C4–cake containing palm oil (control sample). The cake batter was prepared using a four-stage multi-stage mixing approach to improve the aeration (Figure 1). Stage 1 consisted of the creaming and aeration, stage 2 of the ingredient mixing, stage 3 of the baking and cooling, and stage 4, the packaging and storage. It is worth mentioning that prior to the packaging, the cakes were sprayed with an anti-fungal agent. The anti-fungal agent consisted of 400 mL extra hydrated neutral alcohol, 100 mL deionized water, 1.35 g citric acid, 4.25 g sorbic acid, and 3.75 g propylene glycol.

### 2.6. Cake Batter Evaluation

#### 2.6.1. pH

The pH value of the cake batter was determined using a pH meter (model 827; Metrohm, Switzerland). For the pH measurement, 10 g of cake batter was diluted in 100 mL of distilled water at 25 °C [27].

#### 2.6.2. Apparent Density

The cake batter bulk density, also called the apparent density, was determined by dividing the mass of batter (g) by its volume (cm^3^). The apparent density was expressed in g^1^ cm^−3^ and carried out in triplicate. 

#### 2.6.3. Rheological Measurements

The cake batter rheological parameters were determined using an AR 1500 ex (TA Instruments, New Castle, DE, USA) using a stainless-steel flat plate (diameter Φ = 40 mm and 2000 µm gap). The flow curve of the shear stress versus the shear rate of 0–100 s^−1^ was used to calculate the apparent viscosity of the cake batter. For the assessment of the viscoelastic behavior of the cake batter, first a stress sweep was performed by analyzing the sample behavior under the application of stress, ranging from 0.01 to 100 Pa at a frequency of 1 Hz to determine the linear viscoelastic region (LVR). Subsequently, the frequency sweeps at 25 °C and a fixed strain value of 0.10 Pa (within the LVR) from 0.01–10 Hz were performed.

### 2.7. Cake Technological Evaluation

#### 2.7.1. Baking Loss

The baking loss (%) during the baking process was determined in triplicate by Equation (2):

(2) $$\mathrm{baking}\;\mathrm{loss}\;\left(\%\right)=\frac{W_{i}-W_{f}}{Wi}\times 100$$

where *W_i_* is the batter weight before baking; and *W_f_* is the baked cake weight after cooling at room temperature.

#### 2.7.2. Cake Specific Volume

The cake specific volume (cm^3^·g^−1^) was calculated by dividing the cake volume by its mass. The cake volume (cm^3^) was measured in triplicate by millet seed displacement and the mass (g) was measured, using a semi-analytical balance as in [27] on the first day of storage.

#### 2.7.3. Cake Symmetry Index

The cake symmetry index was calculated according to [16]. In short, the height in the center of the slice was computed on three slices situated, respectively, at one-quarter (B), one-half (C), and three-quarters (D) of the cake length, and the symmetry index was calculated on the first day of storage, according to Equation (3):

Symmetry index = 2 × C − B − D(3)

#### 2.7.4. Cake Structure, Appearance, and Color

The images were taken on the first day of storage, using a scanner (PIXMA G2110, Canon, Tokyo, Japan) to analyze the crumb structure and appearance. The color of the cake crumb and crust was assessed by instrumental colorimetry (UltraScan VIS; Hunterlab, Reston, VA, USA), in which the parameters of the color system (L*, a*, b*) were obtained in sextuplicate.

#### 2.7.5. Crumb and Crust Moisture Content

The cake samples, consisting of 3 g from the top, middle, and bottom portions of the differently formulated cakes, were placed in a moisture analyzer (model MOC63u; Shimadzu, Japan) and dried at 105 °C for 20 min. The moisture content was determined on days 1, 7, and 14 of storage and the results were expressed in %, wet basis.

#### 2.7.6. Crumb Instrumental Texture

The texture, measured as the parameter firmness, was carried out in nine replicates and analyzed using a TA.XT2i texture analyzer (Texture Technologies Corp., Scarsdale, NY; USA/Stable Micro Systems, Godalming, England), according to the AACC method 74–09 [28], with slight modifications. One central slice, 25 mm thick, was crushed to 40% of its initial height using a cylindrical aluminum probe of 35 mm diameter (P 35/R). The operational parameters were as follows: 1.7 m/s test speed; 5 g force; and 5 s counting cycle, measuring the compression force.

#### 2.7.7. Water Activity

The water activity (aw) was measured in triplicate in a digital water activity meter (Aqualab 4TE series; Decagon, Pullman, WA, USA) at 25 °C on days 1, 7, and 14 of storage.

### 2.8. Cake Sensory Evaluation

The sensory analyses of the cake formulations were carried out by 120 untrained panelists (74 females, 44 males, and 2 did not reply). The panelists were instructed to evaluate the coded samples based on the attributes of color, aroma, taste, texture, and overall impression on a 9-point hedonic scale where: 1 = disliked extremely; 2 = disliked a lot; 3 = disliked moderately; 4 = disliked lightly; 5 = neither disliked or liked; 6 = liked lightly; 7 = liked moderately; 8 = liked a lot; and 9 = liked extremely, as in [27]. Filtered tap water and a cracker were provided to the panelists to rinse their mouth in between the evaluations. To measure the panelists’ intention to purchase the products, a 5-point scale was used, where: 1 = certainly wouldn’t buy; 2 = probably wouldn’t buy; 3 = not sure; 4 = probably would buy; and 5 = certainly would buy. The answers were expressed as percentages. In addition, the panelists were asked to reply how frequently they consume pound cake. The project was approved by the Ethics Committee of the University of Campinas (CEP-UNICAMP) under the protocol code CAAE 56449522.9.0000.5404/5.318.138.

### 2.9. Statistical Analysis

The results are shown as means ± standard deviation and were analyzed by one factor analysis of variance (ANOVA) and Tukey’s test (*p* < 0.05), using Statistica 8.0 software (Stat Soft. Inc., Tulsa, OK, USA). The graphs were obtained with Microsoft Excel Office 2016.

## 3. Results and Discussion

### 3.1. CA Characterization

#### 3.1.1. Physicochemical Characteristics

The pH value of the CA in this study was 6.38 ± 0.01 and is in accordance with the natural pH values of CA found in other studies, which were also around pH 6 [20]. The slight acidity of CA is associated with its composition consisting of small amounts of organic acids, such as acetic acid, lactic acid, citric acid, succinic acid, and malic acid [12]. In the study of Buhl et al. [15], the authors analyzed the emulsifying properties of CA by the emulsifying activity index (EAI) and the emulsifying stability index (ESB) of the emulsions prepared with the canned aquafaba at various pH values (i.e., 3, 4.5, 6, 7, and 8.5) and reported that the best EAI and ESB was at pH ≥ 6. This indicates that the natural pH of CA is suitable for preparing stable CA-based emulsions, which was confirmed in the study of [14].

**Figure 2:**
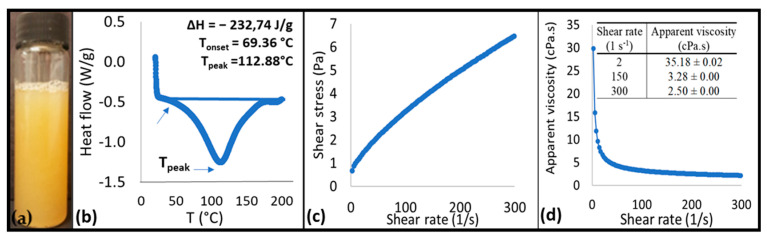
Appearance of CA (**a**); differential scanning calorimetry (DSC) thermogram (**b**); flow curve plotted as shear stress versus shear rate (**c**); and apparent viscosity versus shear rate (**d**) of CA.

The density of the CA in the present work was 1.02 ± 0.01 g mL^−1^ and is also in accordance with other studies, such as 1.02 g mL^−1^ in [20], 1.03 g mL^−1^ in [21], and 1.01 g mL^−1^ in [16]. The total soluble solids (TSS) and the total solids of the CA in the present work were 6.3 ± 0.2 °Bx and 5.7 ± 0.2%, respectively. In the study carried out by [29], the TSS content of the CA varied from 5.09–5.89 °Bx, and the authors reported a direct relation between the increase in soaking time and TSS, because of the accumulation of the water-soluble materials in the aquafaba. A longer cooking time increased the leakage of the water-soluble elements and pigments, which in turn manifested on the overall color of the CA [30]. The color parameters in this study were 16.03 ± 6.22, 0.19 ± 0.15, and 8.59 ± 0.26 for L*, a*, and b*, respectively. In the study carried out by [30], who evaluated different chickpea seeds (CS): the water (W) ratios were 1:2, 1:4, and 2:3 and the cooking times were 15, 30, 45, and 60 min; the L* values ranged from 14.3 to 20.1, a* from 0.1 to 4.1, and b* from 10.7 to 17.5. The CA appearance can be visualized in Figure 2a. In the study of [30], the total solids varied from 3.5 to 15%, and no correlation was reported among the CS:W ratio, color parameters, and total solids. Yet, it is expected that the color variations in CA occur due to many processing conditions, such as the CS:W ratio, temperature, and the soaking and cooking times, as well as the variation in composition among the different varieties of chickpea (i.e., Kabuli and Desi type). In turn, these conditions also play a direct role on TSS, total solids, and the rheological properties (i.e., apparent viscosity) of CA.

#### 3.1.2. Rheological Properties

The shear stress and shear rate parameters (Figure 2c), were adjusted to the power law model (R^2^ = 0.999). The flow behavior index (*n*) value, that shows the level of non-Newtonian traits of the sample, was 0.62 ± 0.04 (*n* < 1), corresponding to a shear thinning pseudoplastic behavior. This behavior can be seen in Figure 2d, where there is a decrease in the apparent viscosity under increased shear strain. The flow consistency index (k) value, which is associated to the sample viscosity, was 0.20 ± 0.07. 

In the study of Meurer et al. [21], the viscosity of the ultrasound-treated CA was around 4.9 cPa·s^−1^ (measured using a Ford viscosity cup at 25 °C) and 4.8 cPa·s^−1^ [20] (measured using a viscosimeter at ambient temperature using a shear rate of 14.1 s^−1^ and a measuring system 12). In our study, the *k* and *n* values were in accordance with the values stated in [30], where the *k* values ranged from 0.01 to 2.24 and *n* from 0.23 to 0.69. The large variation in the *k* value in the latter study was related to the CS:W ratio (1:2, 1:4, and 2:3) and the cooking times (15, 30, 45, and 60 min) used in the study. The lowest *k* value (i.e., 0.01) was obtained at the CS:W ratio of 1:4 and cooking time of 15 min, whereas the highest *k* value (i.e., 2.24) was obtained at the CS:W ratio of 2:3 and cooking time of 60 min.

These results clearly show the influence of increasing the concentration of solids (obtained at higher CS:W ratios and longer cooking times) on the *k* value, and demonstrates the direct role that the cooking conditions play in the rheological properties of CA. Yet, it is still a remaining challenge to make a direct comparison between the studies, as the processing conditions to obtain CA vary greatly among the different studies. It is worth mentioning that not only the CS:W ratio and cooking time affect these parameters, but also soaking time, temperature, as well as the equipment and its operating conditions (e.g., geometry and temperature) used in the measurement.

#### 3.1.3. DSC

The gelatinization peak was not found either in our study or in that of [26], which means that the samples were already gelatinized, probably due to cooking. To produce the aquafaba, the chickpea seeds are cooked in water at temperatures above 100 °C; therefore, most of the starch already undergoes the gelatinization process, which explains the absence of a gelatinization peak [26]. In addition to the lack of gelatinization peak, no denaturation peak (T_d_) was found in our study. However, an event occurred at the temperature of 112.88 °C (Figure 2b). This endotherm could be associated with glass transition [31](and/or the melting of amylose–lipid complexes formed during starch gelatinization) [26,32]. In the study of [32], who produced isolates from pressure-cooked, roasted, soaked, and raw chickpea seeds, an endothermic peak around the same temperature (i.e., 114 to 117 °C) as in this study (i.e., 112.88 °C) was identified, especially for the isolates obtained from the pressure-cooked grains. This behavior was associated with the formation of new conformations of protein aggregates after initial denaturation.

### 3.2. Cake Batter Characterization

#### 3.2.1. Density and pH

The density values of the cake batter ranged from 0.87 to 1.04 g^1^.cm^−3^ (Table 2), and were influenced by the different fat types. In general, the bulk density decreased with the increase in canola oil in the emulsions. This is due to the incorporation of air during the beating of the fat and sugar to obtain the cream. That is, the lower the fat content in the batter, the less air is retained, and consequently, the higher the batter density.

The pH values ranged from 6.6 to 6.8 (Table 2). Ash and Colmey (1973) apud [27], reported that the pH values (i.e., 6.50 to 7.70) are deemed as being good for cake batters, as the pH is related to the texture and color of the cakes.

#### 3.2.2. Rheological Properties

The batter viscosity indirectly affects the cake volume and texture parameters, as it influences the development and steadiness of the air bubbles in the batter in the course of the baking process. Therefore, it is paramount to determine the cake batter viscosity, as it directly affects the quality of the baked cakes [33].

**Figure 3:**
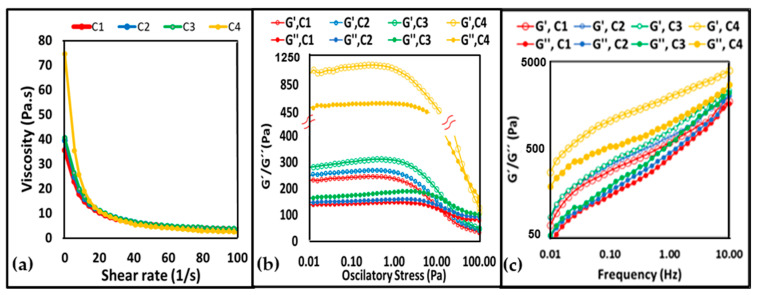
Flow curves plotted as apparent viscosity versus shear rate (**a**); stress sweeps (**b**); and frequency (**c**) tests of C1, C2, C3, and C4 at 25 °C. C1, C2, and C3 contained fats E1 (35% CA and 65% CO), E2 (30% CA and 70% CO), and E3 (25% CA and 75% CO), respectively, and control (C4) contained palm oil.

In this study, C4 had the highest apparent viscosity and σ_0_, followed by C3, C2, and C1. The addition of the different fat types to the pound cake formulation had a significant effect on the batter’s apparent viscosity and the initial shear stress (σ_0_) (Table 2). Moreover, all of the cake formulations showed shear-thinning behavior (Figure 3a).

In a previous study from our research group, the emulsions containing the same amounts of CA and canola oil as in the present study (i.e., E1, E2, and E3, containing 35%, 30%, and 25% of CA, respectively) had an increased apparent viscosity and σ_0_ with the rise in the oil concentration [14]. In the present study, it is possible to observe that this pattern was maintained and that the cake batters containing emulsions with a higher oil concentration also had a higher batter apparent viscosity and σ_0_. The results show that the higher emulsion apparent viscosity leads to a higher viscosity of the cake batter, which indicates that emulsions were able to withstand the vigorous mixing conditions used for the batter preparation.

The G′ value of C4 was the highest (~1070 Pa) under the low-stress values (i.e., 0.01–1 Pa), showing a greater elastic behavior when contrasted to the formulations containing the emulsions, which showed G′ of ~300 Pa, 260 Pa, and 245 Pa, for C3, C2, and C1, respectively (Table 3 and Figure 3b). At the higher stress values (i.e., >1 Pa), the limiting value of the oscillatory stress (OS_L_) values could be identified, which were 2.95, 3.20, 4.5, and 3.98 for C1, C2, C3, and C4, respectively. Identifying the OS_L_ value is essential, as it defines the limit of oscillatory stress that a sample can resist without the collapse of its structure [34].

The loss-tangent values (tan δ_LVR_), determined by the ratio of G′_LVR_ and G″_LVR_, were in the range of 0.52 to 0.61, which indicates a mostly elastic behavior. The determination of the flow point (G′ = G″) is of high importance, as it specifies the stress at which the first non-linear structural change appears. In the present research, the C4 had a higher oscillatory stress value at the flow point (~53 Pa) compared to C3 (~17 Pa), C2 (~14 Pa), and C1 (13 Pa), implying that it endures structural changes at greater stress values. Figure 3c shows the frequency sweep analysis that was carried out within the LRV (i.e., 0.1 Pa). The (G′) was always higher than G″ for all of the formulations and no crossover point (G′ = G″) was detected. These results indicate that all of the formulations displayed gel-like behavior within the tested experimental range (0.01–10 Hz).

### 3.3. Cake Quality Evaluation

**Figure 4:**
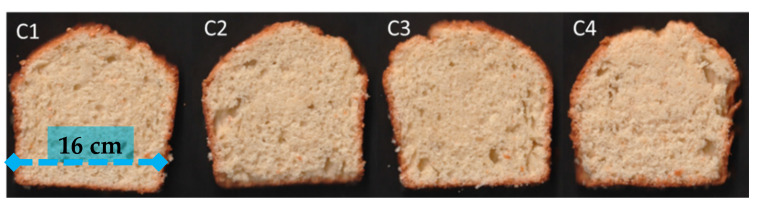
Slices of the cakes made with different fats. C1, C2, and C3 contained fats E1 (35% CA and 65% CO), E2 (30% CA and 70% CO), and E3 (25% CA and 75% CO), respectively, and control (C4) contained palm oil.

The cakes were analyzed during 14 days of storage to verify the effect of replacing PO with the different CA-based emulsions in terms of the quality parameters. Figure 4 shows the appearance of the cakes prepared using different fat types.

The baking loss, specific volume, symmetry index, and color parameters of the cakes made with the different fat types (Table 3) were evaluated 1 day after preparation. No significant difference was observed between the control formulation and the emulsion-based cakes for the parameters of baking loss and symmetry index.

The slight increase in the specific volume of the cakes produced with the emulsions can be attributed to the CA, which possesses the ability to form foams, largely credited to proteins such as albumin [28]. This foaming ability could have led to the higher volume in the cake.

The color parameters (L*, a*, and b*) of the cake crust were not altered by the replacement of palm oil by the emulsions. However, the crumb color parameters of the control formulation (C4) differed from the other formulations, that is, there was a reduction in L*, a*, and b* values when replacing the palm oil with the emulsions. The palm oil has a characteristic, slightly yellowish color (information in the datasheet provided by the company), which may have contributed to the higher color values observed in the crumb of the control formulation.

The moisture, texture, and water activity parameters of the different cakes were evaluated during 14 days of storage, as shown in Table 4. The moisture content and firmness (texture) are important quality indicators for bakery products. They are desirable sensory characteristics in cakes, as they are usually related to a smooth product [35]. However, even under controlled conditions, it is almost inevitable to fully prevent moisture loss. As a consequence, the cakes became less fresh and firmer, [25,27,36].

The moisture content on day 1 showed no significant difference amongst all the formulations. Cakes C1, C2, and C4 had the same behavior of moisture loss over the days; but formulation C3 had a small increase in moisture between days 7 and 14. A relevant fact is that the cakes prepared with the CA-based emulsions had a moisture loss of around 14.5% during the storage period evaluated, while the control (C4) had a 22.8% reduction in moisture, which can contribute to a “drier” cake (which was constantly reported by a panelist in the sensory evaluation, data not shown).

The firmness values on day 1 varied from 851 to 1127 gf amongst the different cakes, and only formulation C1 showed a significant difference from the control. The same behavior was observed on days 7 and 14. An increase in the firmness value was observed on days 1, 7, and 14 of the storage for cakes with different fats. Since the fat is an element that has a positive effect on the texture of cakes, its effect should always be evaluated.

The assessment of the water activity (aw) is a useful tool to predict food stability and safety, as it is linked to microbial growth. A decrease in the crumb aw is normally expected during storage, due to the migration of water from the crumb to the crust [25,37] as observed in our study.

### 3.4. Sensory Evaluation

**Figure 5:**
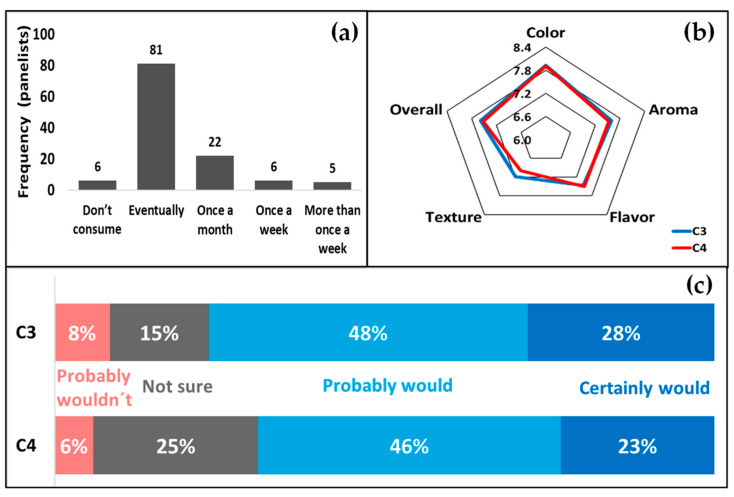
Frequency that the panelists consume pound cake (**a**); response to affective sensory evaluation with respect to the attributes color, aroma, taste, texture, and overall impression (**b**); and purchase intentions (**c**).

As there was no significant difference among C1, C2, and C3 for most of the studied parameters, C3 was chosen to evaluate the sensory acceptance; from a previous study from our research group, the emulsion containing 75% of CO, the HIPE, presented a higher stability as compared to the emulsions containing 70 and 65% of canola oil [14]. Of the 120 panelists, the vast majority (i.e., 114) replied that they consumed pound cake (Figure 5a) and were therefore familiar with pound-cake sensory characteristics. The reported differences between the formulation C3 (HIPE) and C4 (palm oil) were not statistically significant (*p* > 0.05) (Figure 5b). These results are positive, as no significant difference in any of the attributes was noted by the consumers, which indicates that the CA-based emulsions have the potential to replace palm oil in pound-cake recipes, without affecting the sensory attributes.

With respect to the consumers’ purchase intentions, 48% and 28% of the panelists reported they “probably would buy” and “certainly would buy” formulation C3, respectively, whereas for formulation C4, the responses were 46% and 23%. On the other hand, more panelists replied they would probably not buy C3 (i.e., 8%) as compared to C4 (i.e., 6%). Moreover, 25% of the panelists replied they were not sure whether they would or not buy C4, whereas for C3 it was 15%. As for the panelists that would certainly not buy the product, the incidence was 0% for both of the formulations (Figure 5c).

## 4. Conclusions

The replacement of palm oil by CA-based emulsions should not significantly alter the functional characteristics of the pound cake (i.e., cake weight, cake volume and symmetry) and preserve the sensory attributes of the cake as compared to the traditional formula (control). The present study indicated that such a replacement maintained the functional and sensory characteristics of the product, as well as being accepted by the consumers. Therefore, the CA-based emulsions and HIPE can be a suitable replacer for palm oil in the pound cake formulations. In addition, CA is an animal-free food ingredient that can be applied to produce vegan and vegetarian products. Moreover, it is as versatile as other types of vegetable oils, and different oil concentrations can be used to formulate such CA-based emulsions and HIPE.

## Figures and Tables

**Table 1:** Formulation used to prepare the pound cakes.

Ingredients	% (f.b.)	Grams
Wheat flour	95.00	950.00
Corn starch	5.00	50.00
Total flour + starch (f.b. = flour basis)	100.00	1000.00
Fat (palm oil, E1 ^(1)^, E2 ^(2)^, or E3 ^(3)^) ^(4)^	40.00	400.00
Liquid egg ^(4)^	50.00	500.0
Whole milk ^(4)^	45.00	450.00
Refined sugar ^(4)^	78.75	787.50
Baking powder ^(4)^	2.50	25.00
Calcium propionate ^(4)^	0.10	1.00
Salt ^(4)^	0.20	5.00

^(1)^ E1 emulsion consisted of 35% CA and 65% CO ^(2)^ E2 emulsion consisted of 30% CA and 70% CO ^(3)^ E3 emulsion consisted of 25% CA and 75% CO ^(4)^ Ingredients were added based on the weight of f.b., C1, C2, and C3 contained fats E1, E2, and E3, respectively, and control (C4) contained palm oil.

**Table 2:** Density, pH, rheological parameters of apparent viscosity (η) at a shear rate of 5 (η_5_), and the initial shear stress (σ_0_) as determined by steady-shear tests, the storage (G’_LVR_) and loss (G’’_LVR_) moduli at the linear viscoelastic region (LVR), the limiting value of oscillatory stress (OS_L_), the loss-tangent (tan δ_LVR_) at the LVR, the flow-point oscillatory stress (FP_OS_), and the flow-point G (FP_G_) as determined by stress sweep tests (at a 1 Hz frequency) for the batters prepared with different fat types measured at 25 °C.

	Formulation			
Parameter	C1	C2	C3	C4
Density (g^−1^ cm^−3^)	1.04 ± 0.00 ^a^	1.03 ± 0.02 ^a^	1.01 ± 0.00 ^b^	0.87 ± 0.00 ^c^
pH	6.77 ± 0.05 ^a^	6.66 ± 0.04 ^b^	6.60 ± 0.03 ^b^	6.84 ± 0.04 ^a^
η_5_ (Pa.s)	22.87 ± 0.12 ^c^	24.39 ± 0.27 ^c^	26.33 ± 1.21 ^b^	35.25 ± 0.59 ^a^
σ_0_ (Pa)	81.30 ± 6.91 ^c^	84.92 ± 6.31 ^bc^	101.24 ± 7.89 ^b^	163.43 ± 6.64 ^a^
G′_LVR_ (Pa)	245.15 ± 19.04 ^b^	258.65 ± 25.68 ^b^	296.06 ± 7.68 ^b^	1074.10 ± 87.31 ^a^
G″_LVR_ (Pa)	147.40 ± 9.51 ^b^	152.92 ± 8.66 ^b^	176.63 ± 9.09 ^b^	558.66 ± 47.85 ^a^
OS_L_ (Pa)	2.95 ± 0.38 ^b^	3.20 ± 0.60 ^b^	4.50 ± 0.52 ^a^	3.98 ± 0.00 ^ab^
Tan δ_LVR_	0.61 ± 0.02 ^a^	0.60 ± 0.02 ^a^	0.60 ± 0.02 ^a^	0.52 ± 0.00 ^b^
FP_OS_ (Pa)	12.75 ± 1.99 ^b^	14.19 ± 4.08 ^b^	16.88 ± 1.03 ^b^	53.35 ± 3.24 ^a^
FP_G_ (Pa)	123.37 ± 7.10 ^b^	129.36 ± 8.27 ^b^	150.44 ± 17.06 ^b^	233.61 ± 34.69 ^a^

The results (mean value ± standard derivation, *n* = 3) for the same row with different lower-case superscript letters are significantly different based on Tukey’s test at *p* < 0.05.

**Table 3:** Baking loss, specific volume, symmetry index, and color parameters of cakes made with different fats. C1, C2, and C3 contained fats E1 (35% CA and 65% CO), E2 (30% CA and 70% CO), and E3 (25% CA and 75% CO), respectively, and control (C4) contained palm oil.

	Formulation			
Parameter	C1	C2	C3	C4
Baking loss (%)	9.1 ± 0.8 ^a^	9.5 ± 0.5 ^a^	8.9 ± 0.7 ^a^	9.4 ± 0.8 ^a^
Specific volume (cm^3^g^−1^)	2.3 ± 0.0 ^ab^	2.3 ± 0.0 ^b^	2.3 ± 0.0 ^ab^	2.2 ± 0.0 ^a^
Symmetry index	2.6 ± 0.2 ^a^	2.5 ± 0.6 ^a^	2.2 ± 0.6 ^a^	2.5 ± 0.1 ^a^
L*_crust_	49.7 ± 4.0 ^a^	53.9 ± 3.3 ^a^	54.2 ± 6.0 ^a^	55.8 ± 4.9 ^a^
a*_crust_	17.5 ± 1.3 ^a^	16.3 ± 1.1 ^a^	16.3 ± 1.9 ^a^	16.3 ± 2.9 ^a^
b*_crust_	33.0 ± 3.2 ^a^	36.9 ± 2.4 ^a^	35.8 ± 3.1 ^a^	35.8 ± 1.8 ^a^
L*_crumb_	77.8 ± 3.0 ^ab^	77.3 ± 1.9 ^ab^	74.0 ± 4.3 ^b^	79.6 ± 1.2 ^a^
a*_crumb_	0.4 ± 0.4 ^b^	0.7 ± 0.5 ^b^	0.6 ± 0.3 ^b^	1.4 ± 0.5 ^a^
b*_crumb_	21.0 ± 0.6 ^b^	21.5 ± 1.0 ^b^	20.7 ± 1.0 ^b^	23.2 ± 1.2 ^a^

The results (mean value ± standard derivation, *n* = 3, and *n* = 6 for color parameters) for the same row with different lower-case superscript letters are significantly different based on Tukey’s test at *p* < 0.05. For the parameter specific volume (cm^3^ g^−1^), the values of mean ± standard derivation with an additional decimal place are as follows: 2.28 ± 0.02; 2.32 ± 0.04; 2.27 ± 0.03; 2.22 ± 0.02 for C1, C2, C3, and C4, respectively.

**Table 4:** Moisture, firmness, and aw parameters of cakes made with different fats during 14 days of storage.

		Formulation			
Parameter	Day	C1	C2	C3	C4
	1	31.3 ± 0.9 ^aA^	29.7 ± 0.4 ^aA^	29.4 ± 0.5 ^aA^	29.7 ± 2.0 ^aA^
Moisture (%)	7	25.6 ± 0.7 ^bAB^	26.7 ± 0.9 ^bA^	23.0 ± 1.3 ^cB^	23.0 ± 1.3 ^bB^
	14	26.1 ± 0.1 ^bA^	25.7 ± 1.4 ^bA^	25.1 ± 0.4 ^bA^	22.9 ± 0.6 ^bB^
	1	851 ± 116 ^bB^	1164 ± 196 ^cA^	1107 ± 150 ^cA^	1127 ± 98 ^cA^
Firmness (gf)	7	1586 ± 190 ^aB^	1758 ± 302 ^bAB^	1683 ± 121 ^bAB^	1882 ± 220 ^bA^
	14	1896 ± 434 ^aB^	2214 ± 440 ^aAB^	2103 ± 227 ^aAB^	2489 ± 237 ^aA^
	1	0.92 ± 0.00 ^aA^	0.91 ± 0.00 ^aA^	0.91 ± 0.00 ^aA^	0.90 ± 0.00 ^aB^
Water activity	7	0.89 ± 0.00 ^bA^	0.89 ± 0.01 ^bA^	0.89 ± 0.01 ^bA^	0.86 ± 0.00 ^bA^
	14	0.88 ± 0.00 ^cA^	0.88 ± 0.01 ^bA^	0.88 ± 0.00 ^bAB^	0.87 ± 0.01 ^bB^

The results (mean value ± standard derivation, *n* = 3 for moisture and aw, and *n* = 9 for texture) for the same parameter and column with different lower-case superscript letters are significantly different by Tukey’s test at *p* < 0.05. Mean values for the same parameter and row with different upper-case superscript letters are significantly different by Tukey’s test at *p* < 0.05.

## Data Availability

The data used in this study are available in this article.
